# Accurate object localization facilitates automatic esophagus segmentation in deep learning

**DOI:** 10.1186/s13014-024-02448-z

**Published:** 2024-05-12

**Authors:** Zhibin Li, Guanghui Gan, Jian Guo, Wei Zhan, Long Chen

**Affiliations:** Department of Radiation Oncology, The First Affiliated Hospital of Soochow University, Suzhou, China

**Keywords:** Esophagus, Automatic segmentation, Deep learning, Object localization

## Abstract

**Background:**

Currently, automatic esophagus segmentation remains a challenging task due to its small size, low contrast, and large shape variation. We aimed to improve the performance of esophagus segmentation in deep learning by applying a strategy that involves locating the object first and then performing the segmentation task.

**Methods:**

A total of 100 cases with thoracic computed tomography scans from two publicly available datasets were used in this study. A modified CenterNet, an object location network, was employed to locate the center of the esophagus for each slice. Subsequently, the 3D U-net and 2D U-net_coarse models were trained to segment the esophagus based on the predicted object center. A 2D U-net_fine model was trained based on the updated object center according to the 3D U-net model. The dice similarity coefficient and the 95% Hausdorff distance were used as quantitative evaluation indexes for the delineation performance. The characteristics of the automatically delineated esophageal contours by the 2D U-net and 3D U-net models were summarized. Additionally, the impact of the accuracy of object localization on the delineation performance was analyzed. Finally, the delineation performance in different segments of the esophagus was also summarized.

**Results:**

The mean dice coefficient of the 3D U-net, 2D U-net_coarse, and 2D U-net_fine models were 0.77, 0.81, and 0.82, respectively. The 95% Hausdorff distance for the above models was 6.55, 3.57, and 3.76, respectively. Compared with the 2D U-net, the 3D U-net has a lower incidence of delineating wrong objects and a higher incidence of missing objects. After using the fine object center, the average dice coefficient was improved by 5.5% in the cases with a dice coefficient less than 0.75, while that value was only 0.3% in the cases with a dice coefficient greater than 0.75. The dice coefficients were lower for the esophagus between the orifice of the inferior and the pulmonary bifurcation compared with the other regions.

**Conclusion:**

The 3D U-net model tended to delineate fewer incorrect objects but also miss more objects. Two-stage strategy with accurate object location could enhance the robustness of the segmentation model and significantly improve the esophageal delineation performance, especially for cases with poor delineation results.

**Supplementary Information:**

The online version contains supplementary material available at 10.1186/s13014-024-02448-z.

## Background

In the past decade, great progress has been made in the field of deep learning, leading to its widespread application in radiotherapy where it performs tasks such as delineation of the clinical target volume and organs at risk (OARs), previously done by radiologists manually. Automatic segmentation based on deep learning could liberate radiologists from the tedious and repetitive work [1]. On average, the time for manually delineating OARs is about 30 to 90 min, while that for automatic delineation based on deep learning is less than 2 min [2].

Recently online adaptive radiotherapy has been introduced into clinical application [3, 4], in which the image acquisition, target and OARs delineation, radiotherapy plan adjustment, and treatment delivery are performed sequentially while the patient stays stationary on the treatment bed. This process needs to be completed as quickly as possible for the comfort of the patient and the accuracy of the treatment. Therefore, it is necessary to improve the accuracy of the automatic delineation of target volumes and OARs to reduce the time spent manually modifying the delineation.

To continuously improve the accuracy of the automatic delineation and reduce the time required for the radiologist’s manual modifications, various deep learning models have been proposed and continuously improved [5–11].

Currently, multiple studies have focused on all the OARs in the treatment site and even the whole-body OARs, in which all OARs were automatically delineated by a single deep learning model, and satisfactory results were achieved with an average dice coefficient of 0.95 [12]. However, it is a challenging task to delineate small-size OARs due to the sample imbalance problem. The segmentation model tends to focus more on the background and large-size OARs since they have an advantage in terms of pixel counts, which often lends to the under-segmentation of small-size OARs [13]. To solve this class imbalance problem, various new cost functions have been proposed to weaken the contribution of the domain class such as dice loss [9], focal loss [14], and unbalance loss functional [13], which have been proven to be effective. From another perspective, Yunhe proposed a two-stage deep learning network for head-and-neck small-size OARs automatic segmentation, in which the small-size OARs were localized first and smaller images were cropped for accurate image segmentation [8]. Subsequent studies based on this strategy mainly apply different segmentation networks for OARs with different sizes. However, the effect of object location accuracy on segmentation performance was not explored [15].

For thoracic OARs, the average dice coefficient of automatic delineation reaches 0.98, 0.95, 0.90, and 0.86 for the lungs, heart, spinal cord, and trachea, respectively [12, 16, 17]. Nevertheless, caused by the low soft contrast, small size, and large shape variability, the dice coefficient of the esophagus varied greatly from study to study, ranging from 0.49 to 0.84 [16–22]. Similarly, the 95%HD of automatic delineation reaches 2.35, 4.60, 1.64, and 3.48 for lungs, heart, spinal cord, and trachea, respectively, while ranging from 5.18 to 7.16 for esophagus [16–22]. The unsatisfactory delineation requires radiologists to spend a significant amount of time on manual modifications and seriously hinders the clinical application of esophageal automatic segmentation.

Therefore, in this study, we focus on the esophagus automatic delineation based on the classic U-net and 3D U-net models, and apply a two-stage strategy, localizing the object first and then performing automatic delineation, to mitigate the effect of class imbalance. We aimed to improve the performance of esophagus segmentation in deep learning by applying a strategy that involves locating the object first and then performing the segmentation task. At the same time, the effect of the accuracy of object localization on the delineation performance was also evaluated. Finally, a detailed clinical evaluation of the segmentation results was carried out to summarize the performance of the deep learning-based automatic esophageal segmentation.

## Methods

### Training and test cases

For reproducibility and comparability of the results, two public datasets with a total of 100 cases were used in this study. Of these, 60 cases were from the AAPM Lung CT Segmentation Challenge 2017 dataset [22], and 40 cases were from the SegTHOR dataset [23]. Both datasets contain entire 3D thoracic CT images and esophagus delineated by experts. The images are all 512 × 512 pixels for each slice and the in-plane resolution varies between 0.90 mm and 1.37 mm per pixel.

In image preprocessing, the image intensity values were truncated to the range of [-160, 240] to enhance the image contrast, and then the images were normalized to have zero mean and unit variance. All images were resampled to a 0.97 × 0.97 mm in-plane resolution and reformatted into a standard orientation to maintain data consistency.

To avoid potential biases in the model due to small training sample data sets, 5-fold cross-validation was used in this study. For each fold, 68 cases were used to train the model, 12 cases were used to validate the model and adjust the model hyperparameters, and 20 cases, never seen by the model during the training and validation, were used for the final test of the model performance.

### Location and segmentation network

**Fig. 1:**
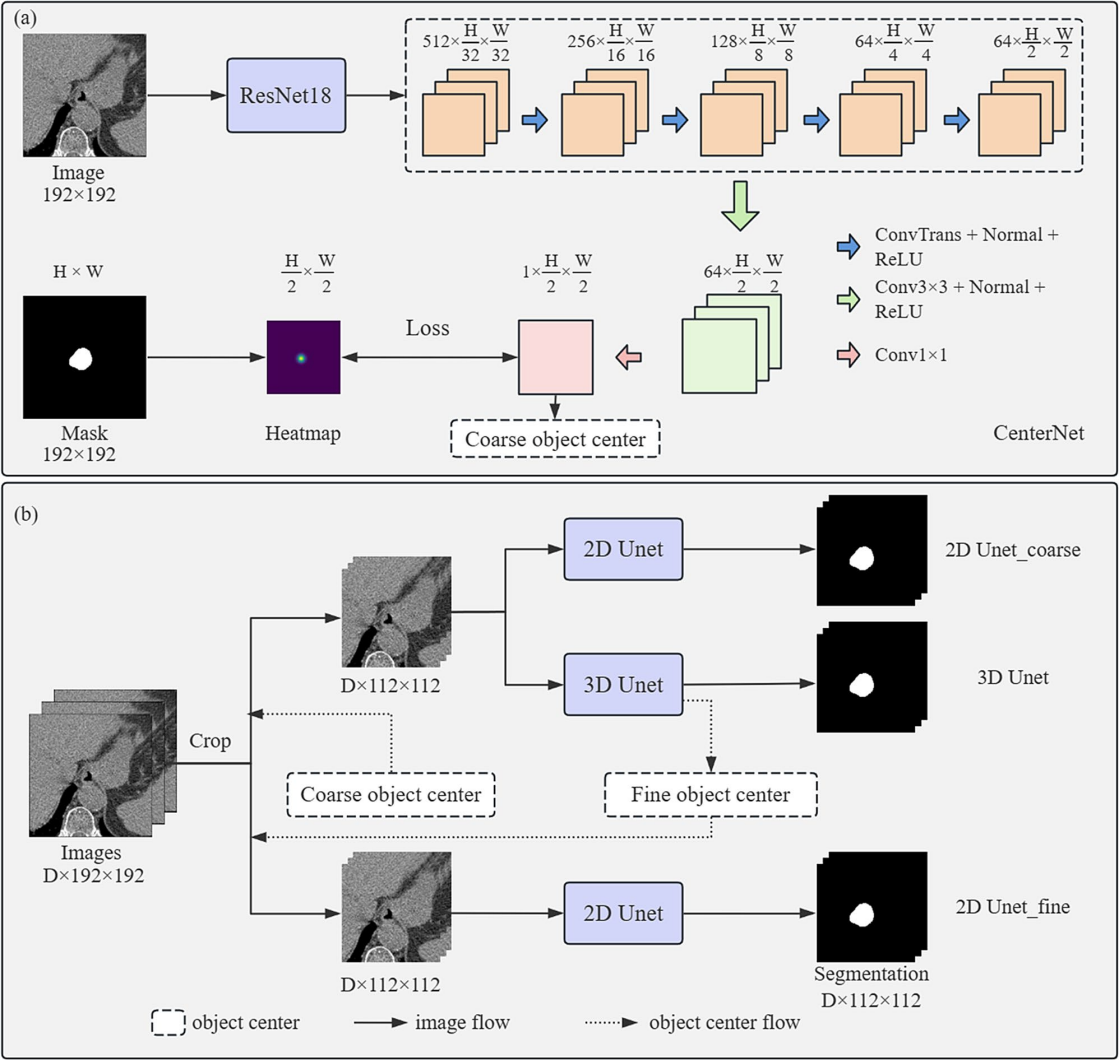
The architecture of the two-stage deep learning framework. It consists of two parts: object location and object segmentation

The entire deep learning framework consists of two parts (Fig. 1). The first part is an object location model, which is a modified CenterNet [24] used to locate the central position of the esophagus first. The second part is a segmentation network used to delineate the esophagus in the cropped image according to the predicted object center.

In the modified CenterNet model, the ResNet18 module [25], a down-sample pathway, was used to extract image features first. The features are gradually recovered through an upsampling pathway to obtain the predicted Gaussian heatmap, and decoding which yields the predicted object center. A supplementary file describes the object location network in more detail [see Additional file 1].

**Fig. 2:**
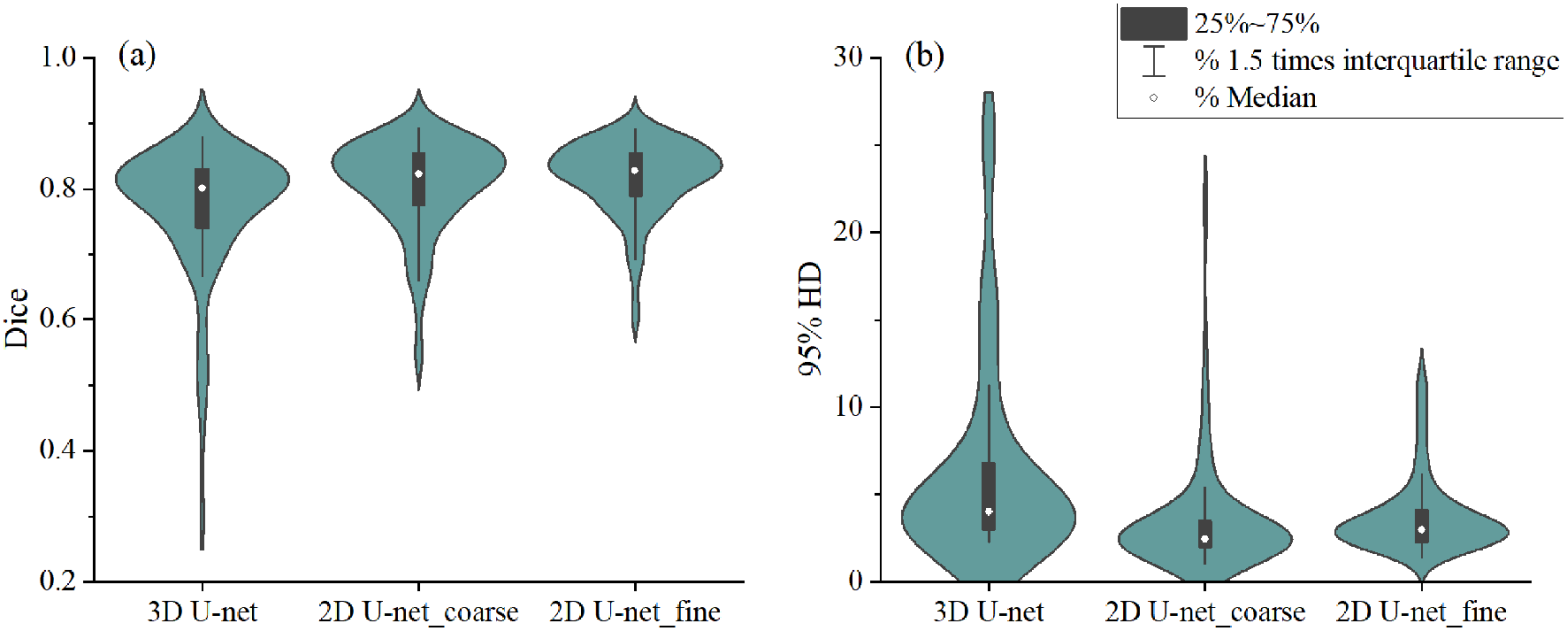
The dice coefficient and 95% HD of esophagus delineated by various segmentation models

**Fig. 3:**
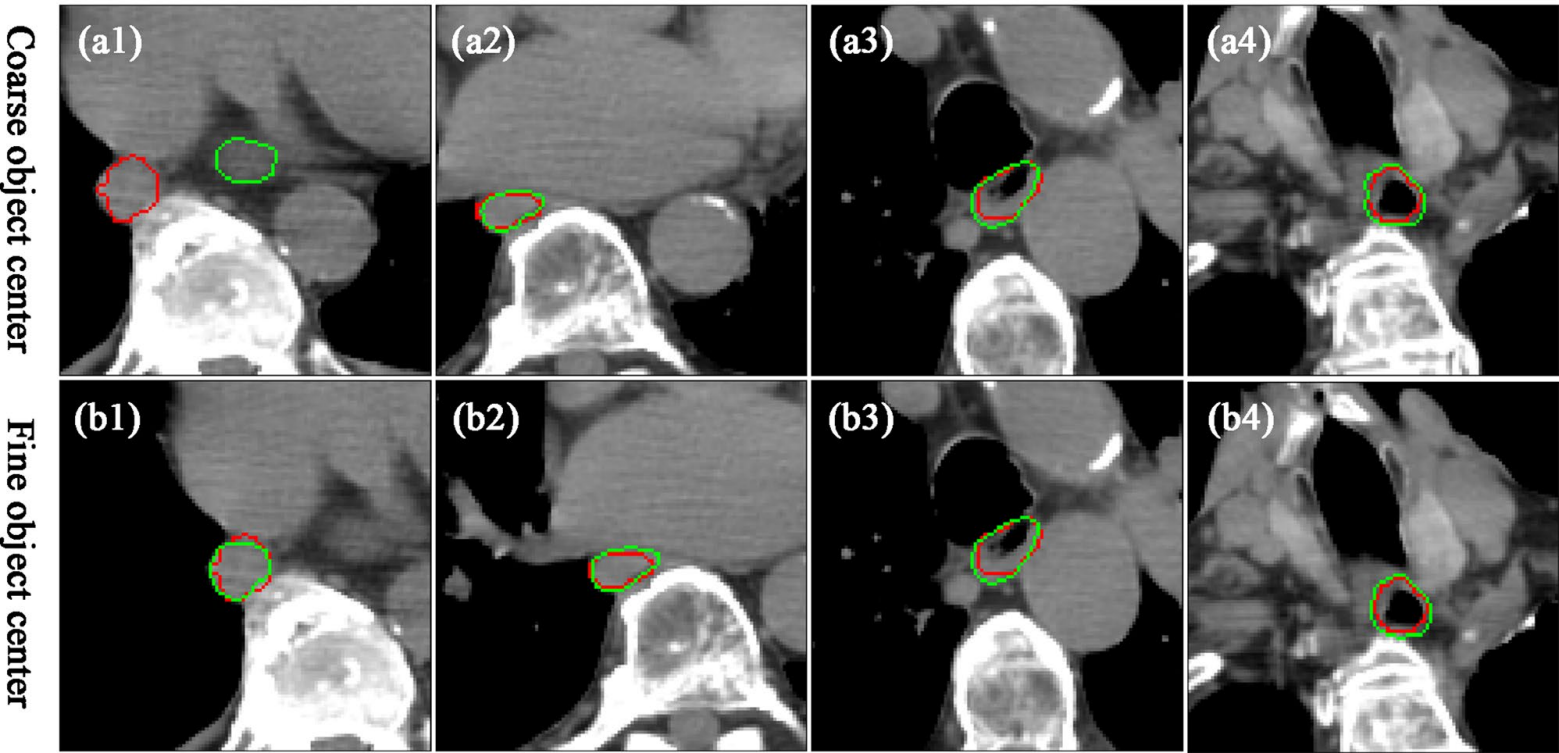
Visualization of a case of esophageal delineated based on the coarse and fine object center. From left to right, they represent different slices of the same case. The red line is marked by experts and the green line is delineated by deep learning models

**Fig. 4:**
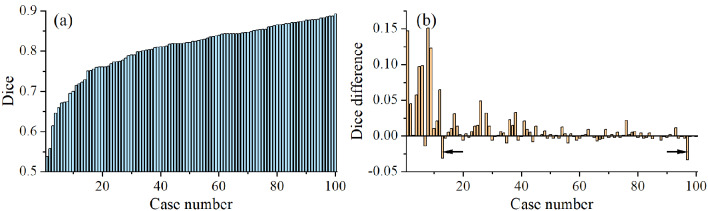
Dice coefficient and the improvement of dice coefficient for all cases. (**a**) the dice coefficient of esophagus delineation based on the coarse object center. (**b**) the improvement in dice coefficient of the model after updating the coarse object center to fine object center. The black arrows indicate exception cases

In the segmentation module, the 2D U-net [17] and 3D U-net [10] models were used to perform esophagus segmentation respectively, and we found that the 3D U-net model performed better in terms of miss delineating the object but the 2D U-net model performed better in terms of identifying boundaries. Therefore, the segmentation was performed using the 2D U-net and the updated object center based on the 3D U-net (See Figs. 2, 3, 4).

For the object location network, the input was a set of center-cropped images with a size of 192 × 192 pixels, and the output was the corresponding Gaussian heatmap. Focal loss was used to optimize the model. For the segmentation network, the input was a set of cropped sub-images with 112 × 112 pixels according to the predicted object center. The model was optimized via a combination loss function as follows:

$${L}_{seg}={L}_{dice}+\alpha {L}_{focal}$$

Where dice and focal represent the dice loss and focal loss, respectively, and the $\alpha$ represents the weight of focal loss, which is adjusted according to the model’s bias. For example, the weight was turned up if the model tended to have fewer predictions.

The deep learning models were implemented based on the Pytorch [26] framework, and all experiments were carried out on a Windows system workstation equipped with the intel core i7-12700 CPU, NVIDIA 4080 GPU, and 32 GB RAM. During training, a set of on-the-fly data augmentation strategies was employed to enhance the model’s generalization ability, including random flip, random rotation within a range of -10 to 10 degrees, random noise, and random crop scaling. The data augmentation and deep learning models training procedures are described in detail in a supplementary file [see Additional file 1].

### Evaluation

For quantitative evaluation, the volumetric dice similarity coefficient was used to evaluate the degree of overlap [27] between the automatic segmentation result and expert delineation, and the 95% Hausdorff distance (95% HD) was used to evaluate the farthest distance between the two delineated boundaries [28]. Besides, the volume ratio was used to evaluate the systematic under or over-segmentation. The quantitative metrics were compared using paired two-sided t-tests.

**Fig. 5:**
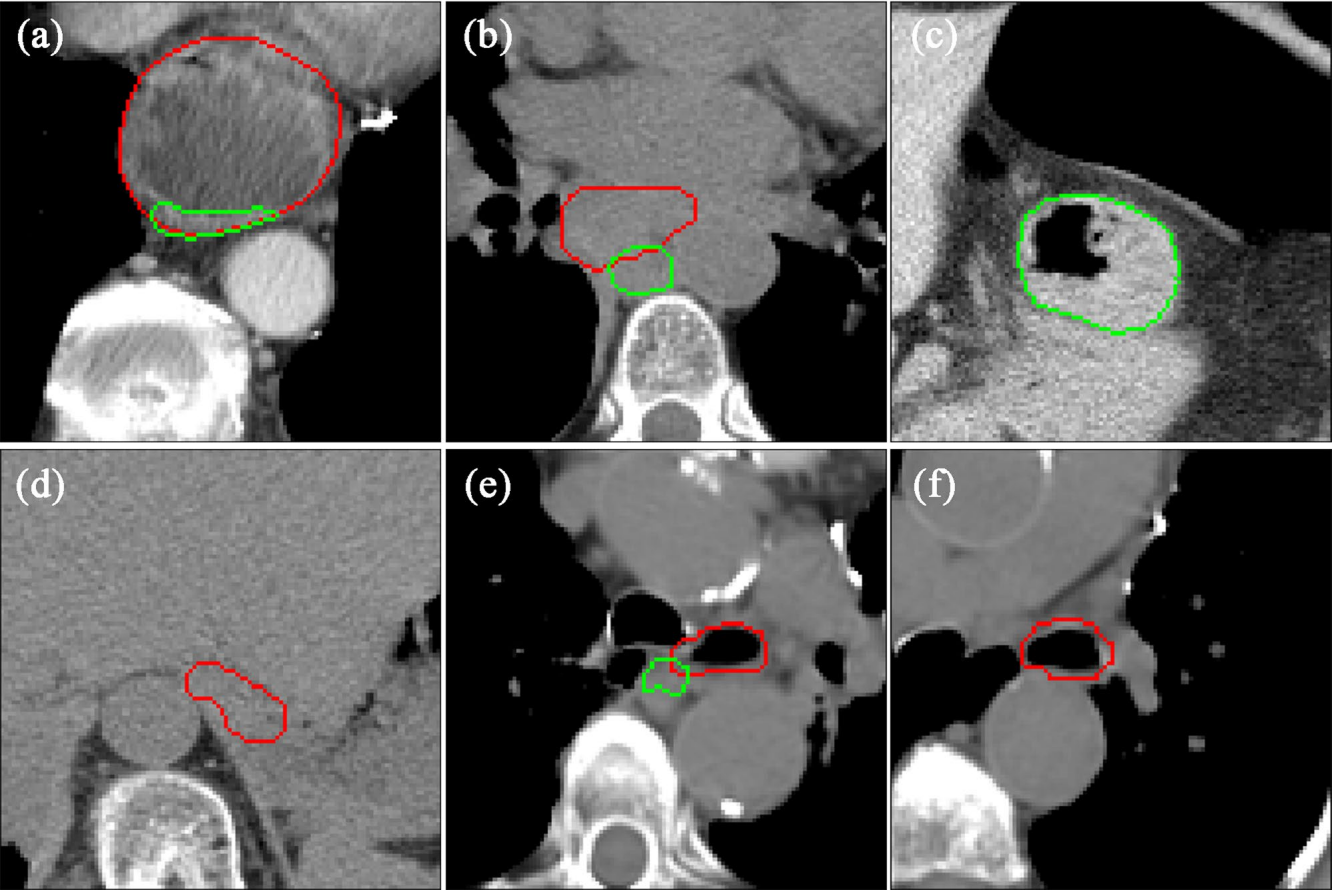
Visualization of esophagus delineation for typical hard samples. The red line is marked by experts and the green line is delineated by deep learning models

In addition, we also focused on the cases with poor delineation performance, namely the robustness issues in clinical applications. Based on the performance of esophagus automatic segmentation in the current study, cases with dice coefficients lower than 0.75 were defined as poor delineation. Using the expert delineation as the standard, each slice was reviewed and analyzed. The phenomenon that there is an expert delineation but no model delineation in a slice is defined as missing delineation (for example, Fig. 5f). The phenomenon that the expert delineation and model delineation were located in different regions (overlap area less than 25% of model delineation) in a slice is defined as the delineation with wrong objects (for example, Fig. 5b). The incidence of delineating wrong objects and missing objects was counted and compared across models using a paired-sample design chi-square test since it is a comparison of sample rates.

Besides, the slice dice coefficients were interpolated to the same length for all cases and then plotted in a graph to evaluate the esophagus automatic segmentation performance in different regions since the esophagus is very long, spanning the neck, chest, and abdomen.

Finally, the total time of object localization and fine segmentation was calculated, to evaluate the feasibility of the proposed model in clinical practice.

## Results

The 2D U-net and 3D U-net segmentation models were trained respectively based on the predicted object center from the modified CenterNet, and the dice coefficient and 95% HD are shown in Fig. 2. The mean dice coefficient for the 2D U-net_coarse segmentation model was 0.807, which was significantly higher than that for the 3D U-net model (*P* < 0.001). Similarly, the average 95% HD for the 2D U-net_coarse segmentation model was 3.566 versus that for 3D U-net with the difference statistically (*P* < 0.001)(See Table 1). In addition, the 2D U-net_coarse model performs better with fewer cases of extremely low dice coefficients.

**Table 1:** Summary of evaluate metrics and *p*-values for all models

models	Dice	95%HD	volume ratio
model1: 3D U-net	0.771	6.55	2.18
model2: 2D U-net_coarse	0.807	3.57	1.42
model3: 2D U-net_fine	0.817	3.76	1.43
*p* (model1 vs. model2)	< 0.001	< 0.001	0.002
*p* (model2 vs. model3)	0.010	0.433	0.752

However, when we reviewed the delineation result for each slice, we found that the incidence of delineating wrong objects is lower in the 3D U-net model compared with the 2D U-net_coarse model (*P* < 0.001) (for example, Fig. 3a1). The frequency of delineating wrong objects was 66 in a total of 10,159 slices for the 3D U-net model, while that value was 92 for the 2D U-net_coarse model. As for the situation of missing objects (for example, Fig. 5f), the frequency was 347, 227 in the total of 10,159 slices for the 3D U-net and 2D U-net_coarse models (*P* < 0.001), respectively.

As described above, the 3D U-net model tends to locate objects more accurately, but it is more prone to missed objects than the 2D U-net. Therefore, the object center was updated according to the prediction of the 3D U-net first, and another 2D U-net segmentation model was trained using the updated object center (fine object center). The dice coefficient for the 2D U-net segmentation model with the fine object center was 0.817, which had significant advantages over that of the model with the coarse object center (*P* = 0.01). The 95% HD was 3.764 and 3.566 respectively for models based on the fine and coarse object center, without a statistical difference (*P* = 0.433). The median volume ratio was 1.17, 0.80, and 0.94 respectively for the 3D U-net, the 2D U-net_coarse, and the 2D Unet_fine, while the mean values of the above data were 2.2, 1.42, and 1.43 respectively. The frequency of delineating wrong objects and missed objects was 63 and 183 respectively in a total of 10,159 slices for the 2D U-net model based on the fine object center, which were both lower than those of the models trained based on the coarse object center.

The dice coefficient of each case for the 2D U-net model based on the coarse object center is shown in Fig. 4a, and the improvement of the model in the dice coefficient after updating the coarse object center to the fine object center is shown in Fig. 4b. The lower the dice coefficients of the case, the greater the dice coefficient improvement after updating the coarse object center to the fine object center. After using the fine object center, the average dice coefficient was improved by 5.5% in the cases with a dice coefficient less than 0.75, while that value was only 0.3% in the cases with a dice coefficient greater than 0.75. In other words, the segmentation models based on the fine object center could improve the delineation performance, especially for some cases with low dice coefficients. However, there were still two cases that performed worse after using the fine object center compared with the coarse object center. Upon reviewing the delineation for each slice in both two cases, we found that there were several slices with over-delineation in the lower boundary of the esophagus (Fig. 5c).

From a clinical perspective to insight into the improvement of dice coefficient caused by fine object location, a case with a large improvement in dice coefficient is shown in Fig. 3. We found that wrong objects were delineated at several slices with large location deviations, while no significant difference was found in the other slice.

It is also important to note that there are still 5 cases out of the 100 cases with a dice coefficient below 0.7, although the 2D U-net segmentation performance improved after using the fine object center. Upon reviewing all cases with poor segmentation performance, we found that there is a large shape variability in some slices, as shown in Fig. 5, including huge esophagus, esophagus with a large cavity, and low contrast with the surrounding tissue. It is prone to delineate wrong objects or miss the delineation in these slices with huge shape variability or low contrast.

**Fig. 6:**
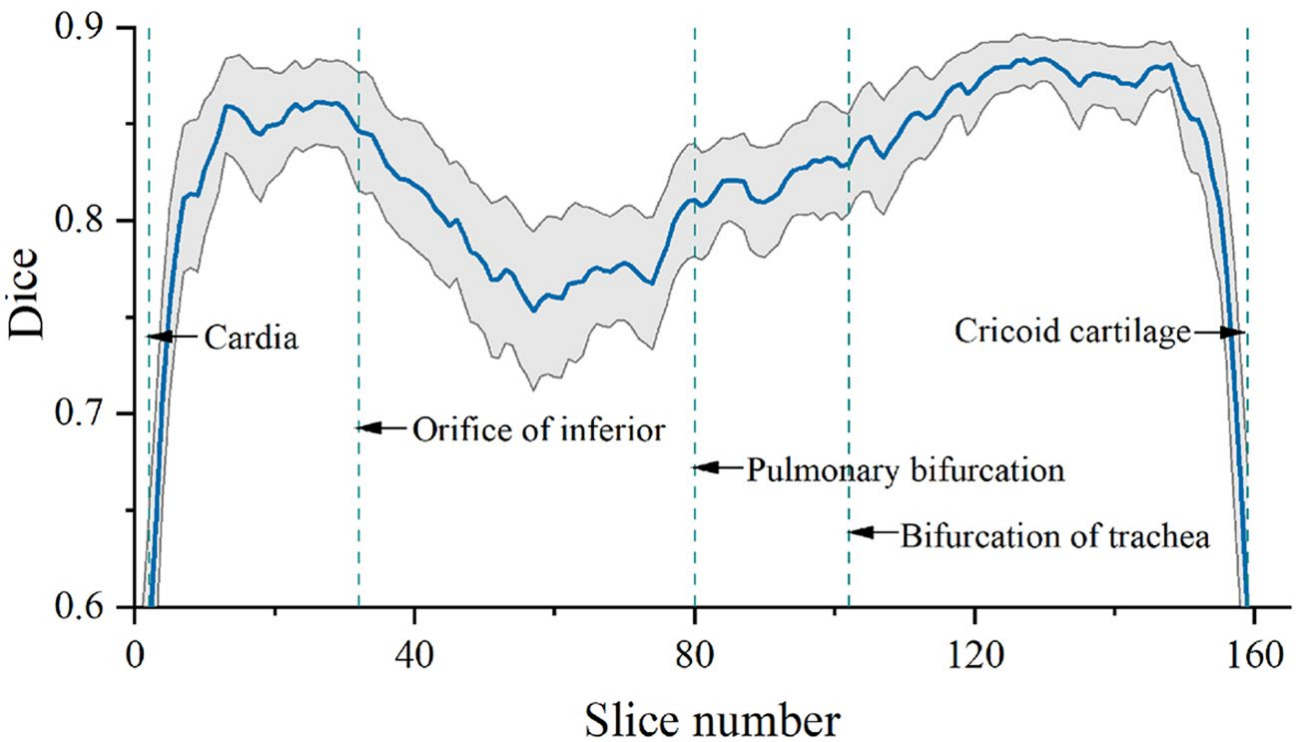
The dice coefficients of esophagus delineation at different slices

Finally, the slice dice coefficients were interpolated to the same length for all cases, and the dice coefficients for different regions of the whole esophagus are shown in Fig. 6. The mean slice dice coefficient was lower at the upper and lower boundaries due to over-delineation or under-delineation of the boundary slices. In addition, the dice coefficients were lower for the esophagus between the orifice of the inferior and the pulmonary bifurcation compared with the other regions.

The time spent for coarse object center localization, fine object center localization, and esophagus segmentation were 33, 30, and 35 s respectively for all 100 cases after the data has been read once, which is achievable in the clinical scenario because multiple OARs need to be segmented. On average, for a 100-slices case, the time taken was 0.96 s to execute the entire localization and segmentation model.

## Discussion

Currently, precise automatic esophagus segmentation remains a challenging task due to its small size, low contrast, and large shape variation. In this work, we adopted a two-stage strategy, focusing on the small-sized esophagus, in which the object center was located first and then the automatic segmentation task was performed using the cropped image based on the predicted object center. We summarized the respective advantages of 3D U-net and 2D U-net models, and we found that accurate object location can improve the performance of segmentation models, and this is obvious for hard sample segmentation. With accurate object location, our model achieved the mean dice coefficients of 0.817 and 95%HD of 3.76 on the esophagus segmentation task. Which is superior to the full-size image model [18] (dice coefficients: 0.770, 95%HD: 5.64) and basic two-stage strategy model [15] (dice coefficients: 0.738, 95%HD: 6.64).

The overall performance of the 3D U-net segmentation model was found to be inferior to that of the 2D U-net model, as evidenced by lower volume dice coefficients and larger 95% HD. Specifically, the 3D U-net model tended to miss objects. The frequency of missing objects was 3.41% and 2.73% in the 3D U-net and the 2D U-net (*p* < 0.001). This may be attributed to the fact that 3D models are more complex, with a larger number of parameters, and require greater computational resources. Therefore, with the same amount of data and limited computational resources, the 3D model may not take full advantage. A similar phenomenon has been observed in several studies. Wenjun et al. trained deep learning models to automatically delineate the abdominal OARs, and the dice coefficients of the esophagus segmented by the 2D U-net were 0.77 and 0.76 in two cohorts, and those were 0.73 and 0.70 for the 3D U-net model [18].

Although the dice coefficient of the 3D U-net segmentation model was lower than that of the 2D U-net model, the incidence of delineating incorrect objects in the 3D U-net model was much lower than that in the 2D U-net model, with the corresponding values of 0.65% and 0.91%, respectively. This may be attributed to the fact that a wider range of contextual features was used in the 3D model, which is beneficial for the model to better understand and make use of the spatial information of objects, such as shape, size, and location [10]. Especially for the esophagus, which is an organ with upper and lower slice continuity, it will be more helpful to identify the esophagus based on upper and lower slice information.

In the 2D U-net segmentation, the model performed better when the input images were cropped using the fine object center compared with using the coarse object center, and the main improvement was the ability to reduce the incidence of delineating wrong objects. This may be attributed to the fact that the contextual information around the object is richer and more symmetrical when the object is in the center of the image, which could provide more adequate information for the model to make predictions. In addition, U-net is a model that uses a symmetric contraction and expansion structure to capture the context information of an image and accurately locate the target [11]. In the down-sampling step, the feature map will be cropped, especially at the edges, therefore the impact of cropping will reduce when the object is at the center of the images.

The phenomenon of sample imbalance is universal, and often causes the class with the disadvantageous sample size to be ignored, resulting in the bias of the model, especially for small-size objects in segmentation tasks. This may be the reason why the dice coefficient of the esophagus automatic segmentation varied greatly among studies [12, 18–20, 22, 29–31]. To address this problem, various loss functions have been proposed to reduce the impact of class imbalance [32–34]. From another perspective, we cropped the useful parts directly from the image for deep learning segmentation, which will directly reduce the rate of class imbalance. And it is a very useful strategy for small-size organ delineation [8]. Although the addition of a separate segmentation model for small-size organs will increase the total automatic segmentation time, about 1 s for an OAR, this time is insignificant compared to the time it takes for the radiologist to manually modify the automatic segmentation results [1].

We also observed that the dice coefficients of the esophagus behind the heart were significantly lower than those for the other sections. This section of the esophagus is adjacent to the heart with numerous surrounding tissues and the contrast between the esophagus and the heart is not obvious, so it is difficult to distinguish the boundary.

In this study, a stable result of esophagus automatic delineation with the mean and median dice coefficients of 0.817 and 0.827 respectively was obtained using the two-stage segmentation strategy and the fine object center. Moreover, this value could reach 0.837 and 0.849 respectively after excluding the influence of upper and lower boundaries on volume dice coefficients.

There are also several limitations in the study. First, there are still some cases of delineation dice coefficient below 0.7, accompanied by the phenomenon of delineating wrong objects or missed delineation. To address these hard cases, it is necessary to add similar samples to the training set to improve the robustness of the models continuously. Second, the dice loss and focal loss were combined with a weight, and the weight was adjusted according to the ratio of the number of pixels that were more or less delineated in the model. It is necessary to research the effect of this weight on the segmentation result carefully in the future. Third, in recent years, the combination of transform and U-net for segmentation tasks has attracted much attention and achieved satisfactory results initially [6, 7]. However, no clear advantage of this combination was observed in our preliminary experiments. Whether small-size OAR could benefit from combining transform models still needs detailed research in the future.

## Conclusions

In summary, we applied the two-stage strategy of localizing the object center first and then performing the segmentation task to delineate the esophagus. Our findings showed that the two-stage strategy could improve the delineation performance of small-size organs, and fine object location could reduce the incidence of poor delineation cases and improve the robustness of models.

### Electronic supplementary material

Below is the link to the electronic supplementary material.

Supplementary Material 1

## Data Availability

The datasets used and/or analysed during the current study are available from the corresponding author upon reasonable request.

## Abbreviations

OAR: Organ at risk
95% HD 95%: Hausdorff distance
AAPM: The American association of physicists in medicine
CT: Computed Tomography
