# Supplementary material for: Accurate object localization facilitates automatic esophagus segmentation in deep learning
Source: Radiat Oncol. 2024 May 12;19:55. doi: 10.1186/s13014-024-02448-z (PMC11088757; doi:10.1186/s13014-024-02448-z)
Supplement: Supplementary file 1 — Supplementary Material 1 [file 13014_2024_2448_MOESM1_ESM.pdf]

Additional file 1

Object location model

The object location model, a modified CenterNet in this study, is used to determine the center of the target organ at risk initially. Although the object center could be determined by preliminary segmentation on a full-size image based on U-net models, the memory footprint and the demand for computing resources in this way are huge.

In the modified CenterNet model, the ResNet18 module, a down-sample pathway, was used to extract image features first. The features are gradually recovered through an upsampling pathway and the predicted Gaussian heatmap was obtained. Finally, the predicted object center is obtained by decoding the predicted heatmap.

To train the modified CenterNet object location model, the object masks need to be converted to the Gaussian heatmap first, in which the value of each pixel represents the probability that it belongs to the object center. The predicted Gaussian heatmap was compared with the real Gaussian heatmap, and the loss was calculated using the focal loss. The Adam optimizer was used to update model parameters. During training, the batch size was set to 8, and the initial learning rate was 1.0e-4. The learning rate is gradually reduced when the training set loss no longer decreases. The training and validation loss curves as well as the prediction error of the object center were shown in Supplementary Fig 1.

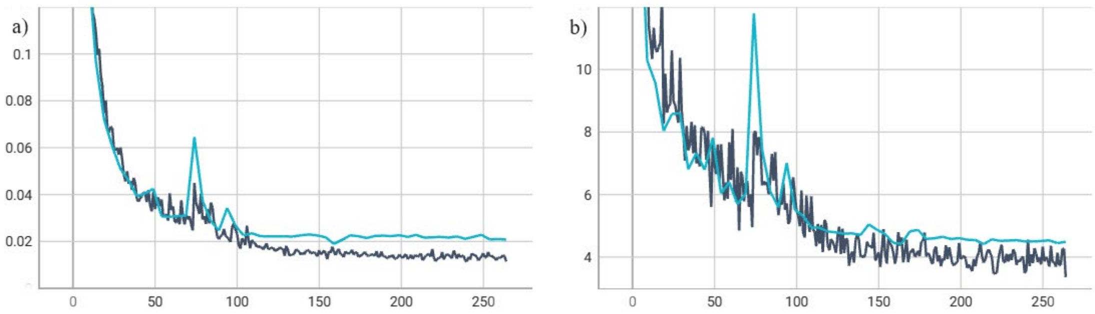

Supplementary Fig 1. The loss curve and prediction error of object centers during training. a) The training and validation loss curves of the object location models. b) the prediction error of object centers. The black curve represents the training set, and the blue curve represents the validation set.

Details on data augmentation

A variety of data augmentation techniques was applied during training, and all the data augmentations were completed on the fly. To increase the diversity of the images, the parameters of data augmentation are randomly selected from the specified range. In the following, x ~ U(a, b) indicates that x was randomly selected from a uniform distribution between a and b. The following data augmentations were applied during training in the given order.

1. Brightness. Voxel intensities were plus x ~ U(-20, 20) in the sample independently to simulate the CT Value deviation between different CTs.
2. Gaussian noise. The Gaussian error with zero mean is added to each voxel in the sample independently. The variance of the Gaussian error is randomly chosen from U(0, 40). After adding brightness adjustment and Gaussian noise, all pixel values are clipped to the original range.

3. Rotation. Each patch was rotated (in degrees) x ~ U(-10, 10) in the plane for both 2D patches and 3D patches to make the image transformation consistent with the real clinical scene. The image rotation is performed in a patch with a size slightly larger than the object patch size to reduce the missing edge data. The same processing is applied for the image masks with the sample parameter.
4. Scale. The image and mask were scaled by x ~ U(0.9, 1.1). The image scale is performed in a patch with a size slightly larger than the object patch size.
5. Flip. Each patch randomly left-right and up-down flipped with a probability of 0.5.

Details on deep learning models training procedures

The SGD optimizer with a momentum of 0.9 was used to update model parameters. The model was optimized via a combination loss function as follows:

$$L_{seg} = L_{dice} + \alpha L_{focal}$$

Where dice and focal represent the dice loss and focal loss, respectively, and the α represents the weight of focal loss, which is adjusted according to the model's bias. For example, the weight was turned up if the model tended to have fewer predictions.

During training, the batch size was set to 16, and the initial learning rate was 1.0e-2. The learning rate is gradually reduced when the training set loss no longer decreases.

The model hyperparameters are adjusted according to the grid search method. In which each hyperparameter was changed in turn during a series of training sessions and the optimal set of hyperparameters was selected based on the result of the validation

set. Finally, the model performance was evaluated using the test set.
